# Targeting of Histone Deacetylases to Reactivate Tumour Suppressor Genes and Its Therapeutic Potential in a Human Cervical Cancer Xenograft Model

**DOI:** 10.1371/journal.pone.0080657

**Published:** 2013-11-19

**Authors:** Dingqing Feng, Jiao Wu, Yuan Tian, Hu Zhou, Ying Zhou, Weiping Hu, Weidong Zhao, Haiming Wei, Bin Ling, Chunhong Ma

**Affiliations:** 1 Key Laboratory for Experimental Teratology of the Ministry of Education, Shandong University School of Medicine, Shandong, China; 2 Anhui Province Key Laboratory of Molecular Medicine, Anhui Provincial Hospital Affiliated to Anhui Medical University, Anhui, China; 3 Department of Obstetric and Gynecology, Anhui Provincial Hospital Affiliated to Anhui Medical University, Anhui, China; 4 Institute of Immunology, Hefei National Laboratory for Physical Sciences at Microscale and School of Life Sciences, University of Science and Technology of China, Anhui, China; 5 Department of Obstetric and Gynecology, China-Japan Friendship Hospital, Beijing, China; University of Pécs Medical School, Hungary

## Abstract

Aberrant histone acetylation plays an essential role in the neoplastic process via the epigenetic silencing of tumour suppressor genes (TSGs); therefore, the inhibition of histone deacetylases (HDAC) has become a promising target in cancer therapeutics. To investigate the correlation of histone acetylation with clinicopathological features and TSG expression, we examined the expression of acetylated H3 (AcH3), RARβ2, E-cadherin, and β-catenin by immunohistochemistry in 65 cervical squamous cell carcinoma patients. The results revealed that the absence of AcH3 was directly associated with poor histological differentiation and nodal metastasis as well as reduced/negative expression of RARβ2, E-cadherin, and β-catenin in clinical tumour samples. We further demonstrated that the clinically available HDAC inhibitors valproic acid (VPA) and suberoylanilide hydroxamic acid (SAHA), in combination with all-trans retinoic acid (ATRA), can overcome the epigenetic barriers to transcription of RARβ2 in human cervical cancer cells. Chromatin immunoprecipitation analysis showed that the combination treatment increased the enrichment of acetylated histone in the RARβ2-RARE promoter region. In view of these findings, we evaluated the antitumor effects induced by combined VPA and ATRA treatment in a xenograft model implanted with poorly differentiated human squamous cell carcinoma. Notably, VPA restored RARβ2 expression via epigenetic modulation. Additive antitumour effects were produced in tumour xenografts by combining VPA with ATRA treatment. Mechanistically, the combination treatment reactivated the expression of TSGs RARβ2, E-cadherin, P21*^CIP1^*, and P53 and reduced the level of p-Stat3. Sequentially, upregulation of involucrin and loricrin, which indicate terminal differentiation, strongly contributed to tumour growth inhibition along with partial apoptosis. In conclusion, targeted therapy with HDAC inhibitors and RARβ2 agonists may represent a novel therapeutic approach for patients with cervical squamous cell carcinoma.

## Introduction

Recent studies have suggested that alteration in the chromatin structure resulting from histone acetylation may be important for the neoplastic process [1]–[3]. The acetylation status of histones is determined by histone deacetylase (HDAC) and histone acetyl-transferase (HAT). Cancer development is driven by genetic and epigenetic changes at the cellular level that activate and inactivate oncogenes and tumour suppressor genes (TSGs), respectively [4]. Transcriptional silencing by histone deacetylation is one of the well-established mechanisms of TSG inactivation [1], [5]–[7]. Some studies have shown that deacetylated histones at promoter regions suppress the expression of TSGs, such as *retinoblastoma* [8], retinoic-acid receptor β (*RARβ*) [9], *p21* [10], *p53* [6], *p16* [11], *E-cadherin* [5], and many others. Thus, the inhibition of HDAC enzymes has been widely accepted as a strategy for modulating gene expression through the induction of histone hyperacetylation and the re-expression of silenced TSGs.

Of all the TSGs, RARβ2, and E-cadherin play an essential role in the modulation of cellular growth and differentiation in both healthy and malignant cells [9]. The ability of retinoic acid to act as a chemopreventive agent is facilitated primarily by its interaction with RARβ2 [9], [12]. E-cadherin is an intercellular adhesion molecule in epithelial cells, which binds to β-catenin to form a complex that plays an important role in maintaining the morphological integrity of the normal epithelium [13], [14]. Studies of epithelial tumours have consistently shown that the downregulation of RARβ2 is a crucial event in malignancy and that the subsequent aberrant expression of E-cadherin/β-catenin complexes correlates with the conversion of early-stage tumours into invasive malignancies [15], [16]. The loss of RARβ2 expression in tumour cells has been attributed to the silencing of the gene promoter region via histone deacetylation and hypermethylation. Therefore, the use of HDAC inhibitors, either alone or in combination with DNA methyltransferase (DNMT) inhibitors, has been shown to restore the transcription and growth regulatory effects of RARβ2 [9], [17], [18]. Our previous work has also revealed that the combination of valproic acid (VPA), a clinically available HDAC inhibitor, and all-trans retinoic acid (ATRA) promotes synergistic effects in reactivating dormant RARβ2 and strongly inhibiting cervical cancer cell growth *in vitro* and *in vivo* by promoting differentiation via the PI3K/Akt pathway [19]. However, to our knowledge, the correlation between histone acetylation and TSG expression in human cervical cancer tissue specimens and the potential exploitation of histone acetylation in targeted cancer therapy have not been fully evaluated. In this study, we investigated histone H3 acetylation and TSG expression in cervical cancer and its association with clinicopathological parameters. Furthermore, we evaluated the therapeutic potential of the HDAC inhibitor VPA combined with ATRA in treating a tumour xenograft model derived from human cervical carcinoma.

## Materials and Methods

### Ethics statement

This study protocol was approved by the Ethics Committee of Anhui Medical University. The paraffin-embedded patient tissue samples used in this study were obtained as described in our previous report [20]. The cancerous tissues for implantation in mice were obtained from patients with cervical cancer. Written informed consent was obtained from each patient. Approval for *in vivo* experiments involving animals was granted by the Committee on the Use and Care of Animals of Anhui Medical University.

### Patients and samples

Formalin-fixed paraffin-embedded samples derived from cervical lesions in 65 patients diagnosed with cervical squamous cell carcinoma were drawn from the archives of the Department of Pathology of Anhui Provincial Hospital affiliated to Anhui Medical University during the period of 2002 to 2008. The clinical stages were determined by two certified gynaecologists according to the modified International Federation of Gynecology Obstetrics (FIGO) system for cervical cancer published in 2000. The tumours were classified as well - moderately, or poorly differentiated - by at least two pathologists according to the criteria proposed by the World Health Organization. Detailed clinicopathological information is shown in Table 1. All patients were treated with radical hysterectomy. None of the patients had received any tumour-specific therapy before the surgical excision.

**Table 1:** Relationship between immunoreactivity of four parameters and clinical variables.

Variables		AcH3				RAR-β2				E-cadherin				β-Catenin			
category	subcategory	0	1–4	>4	*P*-value	0	1–4	>4	*P*-value	0	1–4	>4	*P*-value	0	1–4	>4	*P*-value
Age	≤50 (n = 44)	2	27	15	0.062	7	30	7	1.000	8	22	14	0.070	10	20	14	0.138
	>50 (n = 21)	0	9	12		3	15	3		2	7	12		2	9	10	
Histological differentiation	Good (n = 18)	0	1	17	0.000	0	11	7	0.000	0	5	13	0.000	0	5	13	0.000
	Moderate (n = 27)	0	17	10		2	22	3		4	10	13		5	12	10	
	Poor (n = 20)	2	18	0		8	12	0		6	14	0		7	12	1	
Clinical stage	I (n = 52)	1	30	21	0.889	8	37	7	0.583	7	25	20	0.965	11	23	18	0.287
	II (n = 13)	1	6	6		2	8	3		3	4	6		1	6	6	
Nodal metastasis*	N (-) (n = 51)	0	24	27	0.000	3	39	9	0.001	4	22	25	0.000	4	24	23	0.000
	N (+) (n = 14)	2	12	0		7	6	1		6	7	1		8	5	1	

* N (-), no nodal metastasis; N (+), nodal metastasis.

### Cell culture and treatment

Human cervical cancer cell lines HeLa, SiHa, CaSki, and C33A were purchased from American Type Culture Collection (ATCC) and cultured in DMEM (Gibco, USA) with 10% fetal bovine serum (Gibco). VPA (Sigma-Aldrich, USA) was dissolved in PBS and used at a final concentration of 3 mmol/L. SAHA and ATRA were purchased from Sigma-Aldrich, dissolved in DMSO, and used at final concentrations of 10 µmol/L and 1 µmol/L, respectively. The cells were treated with either VPA or SAHA alone or in combination with ATRA for 48 h. The control cells were treated with the vehicle alone.

### Immunohistochemical staining and evaluation immunoreactivity

Paraffin blocks of the tumours were cut into 5 *µ*m slices and then processed using standard deparaffinisation and rehydration techniques. Endogenous peroxidases were blocked using 3% hydrogen peroxide. For antigen retrieval, the slides were processed by microwave heating in 0.01 M sodium citrate (pH 6.0). The slides were pre-incubated in 5% goat serum in TBS (pH 7.6) before primary antibodies were added. The following primary antibody dilutions were used: 1∶100 for acetylated histone H3 (Santa Cruz, USA), 1∶100 for RARβ2 (Santa Cruz), 1∶50 for E-cadherin (Abcam, UK), 1∶100 for β-catenin (Abcam), 1∶100 for involucrin (Santa Cruz), 1∶500 for loricrin (Abcam), and 1∶200 for Ki67 (Boster, China). The binding of the primary antibodies was visualised using the ChemMate Detection Kit (Boster). The slides were lightly counterstained with Mayer's haematoxylin for 30 seconds.

Immunoreactivity was semiquantitatively evaluated on the basis of staining intensity and distribution using immunoreactivity score as follows: intensity score × proportion score [16], [21]. The intensity score was defined as 0, negative; 1, weak; 2, moderate; or 3, strong, and the proportion score was defined as 0, negative; 1, <10%; 2, 11-50%; 3, 51-80%; or 4, >80% positive cells. The total score ranged from 0 to 12. Immunoreactivity was divided into three levels on the basis of the final score: negative immunoreactivity was defined as a total score of 0; low immunoreactivity was defined as a total score of 1 to 4; and high immunoreactivity was defined as a total score higher than 4. The stained tumour tissues were scored by two researchers who were blinded to the clinical data.

### RNA isolation and real-time PCR quantification

One microgram total RNA, which was extracted from the tissues, was converted to cDNA using Superscript III reverse transcriptase (TaKaRa, Japan). Quantitative real-time PCR (qRT-PCR) assays were performed using 50 ng cDNA, specific primers (Table 2), the SYBR® *Premix Ex Taq*™ Kit (TaKaRa), and an Opticon® 2 real-time PCR instrument (MJ Research, USA). Gene expression levels were determined using the standard curve method and expressed relative to GAPDH expression.

**Table 2:** Specific primer sequences for semi-quantitative and real-time PCR.

Primer name	Sequence (5′-3′)	Product (bp)	Application
RARβ2-RARE-F	CCTCTCTGGCTGTCTGCTTTTG	190	ChIP
RARβ2-RARE-R	CACTTCCTACTACTTCTGTCAC		ChIP
GAPDH-promoter-F	TACTAGCGGTTTTACGGGCG	166	ChIP
GAPDH-promoter-R	TCGAACAGGAGGAGCAGAGAGCGA		ChIP
RARβ2-F	TGGGTAAATACACCACGAA	119	Q-PCR
RARβ2-R	TTTAGCAAACTCCACGAT		Q-PCR
E-cadherin-F	CTACAATGCCGCCATCGCTTACA	131	Q-PCR
E-cadherin-R	GGAAACTCTCTCGGTCCAGCCCA		Q-PCR
Involucrin-F	TCCAGTCAATACCCATCAGG	156	Q-PCR
Involucrin-R	TGCTCACATTCTTGCTCAGG		Q-PCR
Loricrin-F	AGTGGACTGCGTGAAGAC	112	Q-PCR
Loricrin-R	GCCAGAACCGCTGCTACC		Q-PCR
GAPDH-F	CTTAGCACCCCTGGCCAAG	151	Q-PCR
GAPDH-R	GATGTTCTGGAGAGCCCCG		Q-PCR

### Antibodies and immunoblotting

The acetylated histone H3 (AcH3, 1∶500), RARβ2 (1∶500), and involucrin (1∶500) antibodies were purchased from Santa Cruz Biotechnology. The Stat3 (1∶1000), p-Stat3 (1∶2000), P21 (1∶1000), and GAPDH (1∶2000) antibodies were obtained from Cell Signaling Technology. The E-cadherin (1∶500), β-catenin (1∶5000), and loricrin (1∶2000) antibodies were purchased from Abcam. The P53 (1∶500), caspase-3 (1∶1000), and Bcl-2 (1∶200) antibodies were obtained from Boster Technology. Protein was prepared from the tissues according to the kit (#89900, Thermo, USA) manual except for the addition of an acid extraction step for histones [19]. After electrophoresis, the proteins were transferred to polyvinylidene fluoride (PVDF) membranes and probed with the indicated primary antibodies. Incubation with species-specific secondary antibodies (Cell Signaling) was performed at room temperature for 1 hour. The blots were developed with chemiluminescent substrate (Thermo), and autoradiography was performed with X-OMAT film (Kodak, Rochester, NY).

### Chromatin immunoprecipitation assay

Chromatin immunoprecipitation (ChIP) was performed using the EZ-ChIP Assay kit (Millipore, USA) with a ChIP-grade antibody to acetylated histone H3 (H3K9ac, Abcam). Briefly, cervical cancer cells were cultured in 150 mm culture dishes. The cells were then treated with either VPA (3 mmol/L) or SAHA (10 µmol/L) alone or in combination with ATRA (1 µmol/L) for 48 h. The cells were then cross-linked with 1% formaldehyde for 10 min at room temperature, and the reaction was quenched with 0.125 M glycine. The cells were scraped, resuspended in lysis buffer (1% SDS, 10 mM EDTA, and 50 mM Tris-HCl, pH 8.1) supplemented with a protease inhibitor cocktail on ice, and sonicated to obtain DNA fragments of 200–1000 bp in length, as confirmed by electrophoresis on 1% agarose gels. The sheared DNA was centrifuged at 12000×*g* at 4°C for 10 min, and the supernatant was collected. Ten percent of the supernatant was reserved for use as input DNA and processed for further use as a positive control. Soluble chromatin was immunoprecipitated overnight with an anti-H3K9ac antibody. The immunoprecipitated chromatin complex was harvested using protein G-agarose beads, and the crosslink was reversed by adding NaCl to a final concentration of 200 mM at 65°C for 5 h. The DNA was purified using the spin columns provided with the kit. The DNA samples, as well as the input material and the mock immunoprecipitation samples, were used as templates for semi-quantitative and real-time PCR to determine the relative enrichment of the RARβ2-RARE promoter. The primers for the RARβ2-RARE promoter are shown in Table 2.

### Implantation of original human tumour xenografts in mice and drug therapy

Four-week-old female BALB/c nude mice were obtained from the Laboratory Animal Center of the Chinese Academy of Science (Shanghai, China) and maintained in a pathogen-free animal facility for 1 week before use. A cervical cancer specimen of poorly differentiated squamous cell carcinoma, which was confirmed by histology, was obtained with informed consent from a patient undergoing surgery within 1 hour after hysterectomy. The specimen was washed with phosphate-buffered saline (pH 7.4) containing 10,000 U/ml penicillin and streptomycin and then sectioned into small pieces of approximately 2–4 mm^3^ for implantation. These small tissue blocks were implanted into the subcutaneous tissue of three nude mice at the dorsum using a trocar.

After 6–8 weeks, a portion of the tumour nodules that developed was excised under sterile conditions. This specimen was immediately subdivided into small fragments and injected into other nude mice. Once palpable tumours were established, 20 mice were randomly placed into four groups: control, VPA, ATRA, and combination. VPA was diluted in 0.9% sodium chloride, while ATRA was dissolved in purified sesame oil. The mice were administered VPA (300 mg/kg/d) and/or ATRA (15 mg/kg/d) daily via intraperitoneal (i.p.) injection and intragastric (i.g.) gavage needle, respectively. The control group received the vehicle alone following the same schedule. Tumour volume was measured with a calliper twice weekly and calculated according to the following formula: (length×width^2^)/2. The animals were treated for 4 weeks and then sacrificed. Tumors were harvested and fixed with 4% paraformaldehyde for pathologic evaluation or frozen on liquid nitrogen for PCR, ChIP, and immunoblotting analysis just as the tumors mass were recorded. Tumor growth inhibition (TGI) was calculated by subtracting from 100% the mean treated tumor mass/mean control tumor mass × 100%.

### TUNEL assay

Upon the receipt of each tumor specimen, a small portion of the tumor was fixed in 4% paraformaldehyde and embedded in paraffin. After deparaffinization, the sections were washed and permeabilized for 5 min on ice with 0.1% Triton X-100 and then incubated with Proteinase K (Sigma-Aldrich) for 10 min at room temperature. TUNEL staining was performed using an *in situ* apoptosis-detection kit (Keygene Technology, China), according to the manufacturer's directions. The sections were covered with TUNEL labeling reaction mix (equilibration buffer, biotin-11-dUTP, TdT enzyme) and incubated at 37°C for 1 h in a humidity chamber. The sections were further labeled with streptavidin-FITC in the dark for 30 min. After counterstaining with DAPI (2 µg/ml), TUNEL-positive cells were counted from fluorescence images taken from 10 random fields at 200× magnification.

### Statistical analysis

All statistical analyses were performed using the SPSS software package (version 13; SPSS Inc., USA). The correlation between the clinicopathological parameters and immunohistochemical data was assessed using the chi-squared test or Fisher's exact test. The association between the expression of AcH3, RARβ2, E-cadherin, and β-catenin was analysed using the chi-squared test for a linear trend. The inter-observer concordance in the immunoreactivity score between two researchers were assessed by κ-statistics. According to Fleskens [22], κ-values <0.20 indicate poor agreement; 0.21–0.40 fair agreement; 0.41–0.60 moderate agreement; 0.61–0.80 good agreement; and 0.81–1.00 excellent agreement. ANOVA was used to analyse the statistical significance for the relative enrichment of RARβ2 promoter in ChIP assay and the *in vivo* xenograft models. A *P*-value of <0.05 was considered statistically significant.

## Results

### Clinicopathological characteristics

A total of 65 cases of primary cervical squamous cell carcinoma were analysed. Their clinicopathological features, including tumour differentiation, staging information and nodal metastasis, were reviewed and are summarised in Table 1.

### Association of the clinicopathological variables with the expression levels of AcH3, RARβ2, E-cadherin, and β-catenin

**Figure 1:**
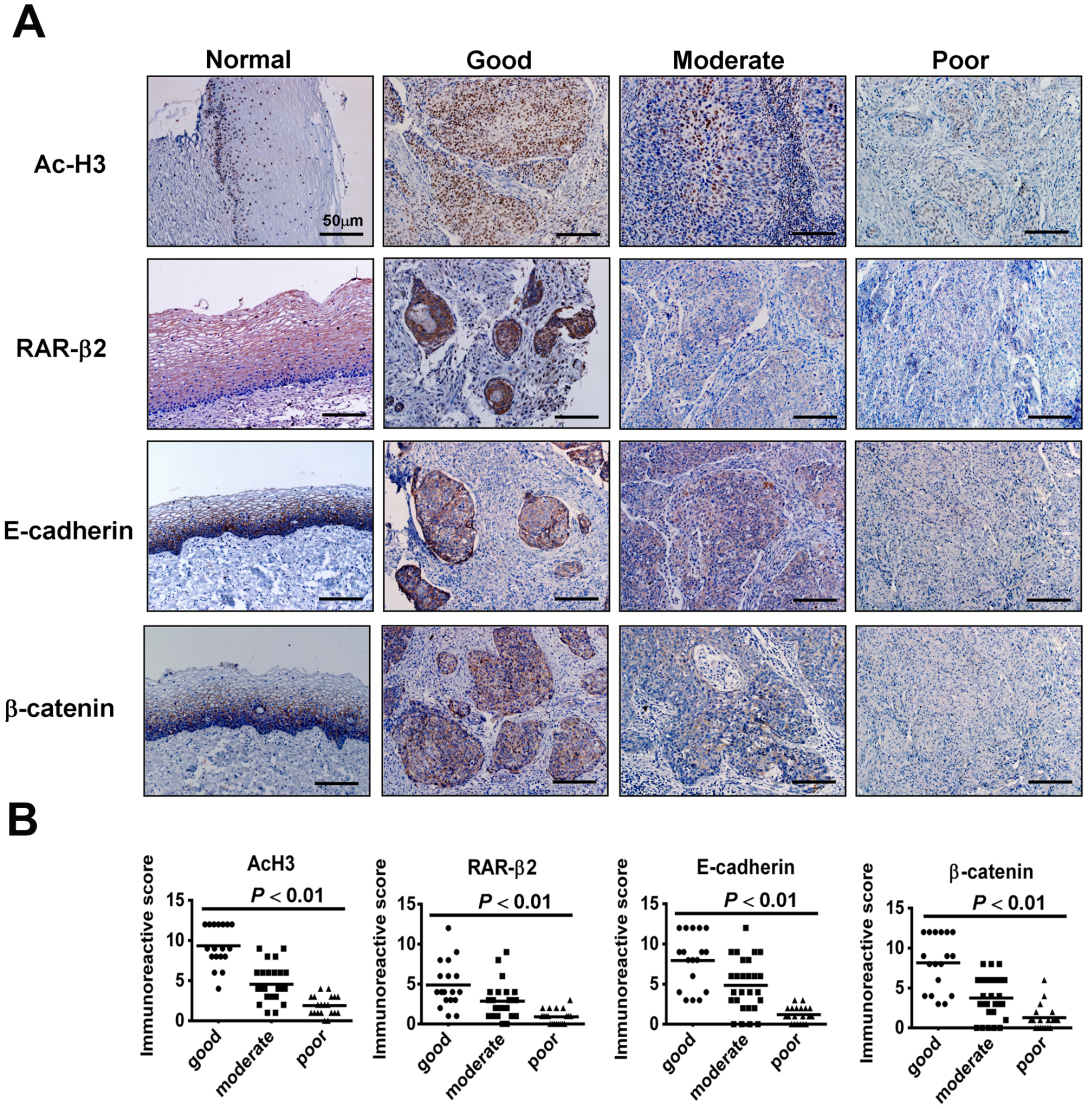
Immunohistochemistry for AcH3, RARβ2, E-cadherin, and β-catenin in cervical cancer tissues. (A) The left panels show the sections from normal cervical tissue. AcH3 staining was strong in the tumour cells in well-differentiated carcinomas. AcH3 expression was reduced or absent in moderately and poorly differentiated carcinomas, respectively. Similar expression patterns were observed for the staining of RARβ2, E-cadherin, and β-catenin. (B) There were significant differences in the immunoreactivity scores for AcH3, RARβ2, E-cadherin, and β-catenin among well, moderately, and poorly differentiated squamous cell carcinomas.

Representative results of our immunohistochemistry analysis for AcH3, RARβ2, E-cadherin, and β-catenin are shown in Figure 1. In the normal cervical epithelium, all four parameters were positively stained in the cells of the suprabasal to the basal layer, with distinct expression of AcH3 in the nuclei, RARβ2 in the cytoplasm and nuclei, and E-cadherin and β-catenin mainly on the membrane (Figure 1). In the cancerous tissue, the immunoreactivity intensity of these four parameters was strong in well-differentiated tumours and reduced in moderately differentiated carcinoma, with weak or negative labelling in poorly differentiated carcinomas (Figure 1A).

Immunohistochemical staining was scored by two researchers. Table 3 shows the concordance between the two researchers for the three levels of immunoreactivity for AcH3, RARβ2, E-cadherin, and β-catenin. A comparison of the results using κ-statistics showed excellent agreement for RARβ2 and E-cadherin scoring (κ = 0.840, 0.876) and good agreement for AcH3 and β-catenin scoring (κ = 0.710, 0.657). The overall κ-value was 0.778 (good agreement), indicating concordance between the immunoreactivity scores provided by the two researchers.

**Table 3:** Concordance between two researchers in the immunoreactivity score.

Immunohistochemistry analysis	κ-value	*p*-value	Strength of agreement
AcH3	0.710	0.000	good
RARβ2	0.840	0.000	excellent
E-cadherin	0.876	0.000	excellent
β-catenin	0.657	0.000	good
Overall	0.778	0.000	good

The relationships between the clinicopathological features and the immunoreactivity scores for AcH3, RARβ2, E-cadherin, and β-catenin are shown in Table 1. There were no statistically significant differences with respect to age or clinical stage between these four parameters. However, the expression of these four parameters showed a significant correlation with histological differentiation (*P*<0.01) and with nodal metastasis (*P*<0.01) (Table 1). The distributions of the positively stained tumour cells plotted against their immunoreactivity scores in the patient samples are shown in Figure 1B. AcH3, RARβ2, E-cadherin, and β-catenin expression correlated positively with histological differentiation (r = 0.737, 0.531, 0.574, and 0.549, respectively, *P*<0.01) (Table 1). For H3 acetylation, 94.4% (17/18) of the well-differentiated cases showed high expression levels, while 37% (10/27) of the moderately differentiated cases showed high expression levels. Strong expression was not observed in the poorly differentiated cases (0/20). The proportion of tumors with high immunoreactivity for E-cadherin and β-catenin expression was over 72% in patients with well-differentiated tumours. However, for RARβ2 expression, only 39% (7/18) of the patients had a total score > 4, while 61% (11/18) of the patients exhibited low immunoreactivity. Similarly, there was a statistically significant correlation between H3 acetylation and the expression of RARβ2, E-cadherin, and β-catenin (r = 0.560, r = 0.731, and r = 0.733, respectively, *P*<0.01) (Figure 1B).

### HDAC inhibitors in combination with ATRA restore RARβ2 expression through RARβ2-RARE

**Figure 2:**
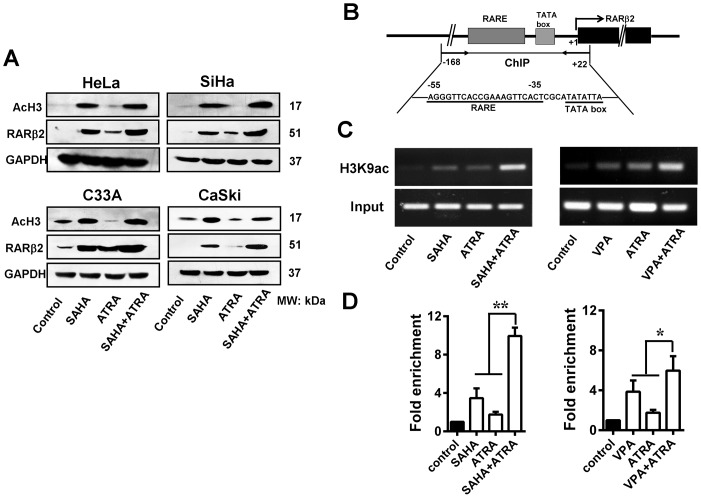
Histone modification at the RARβ2-promoter region induced by HDAC inhibitor and ATRA treatment. (A) SAHA (10 µmol/L) treatment for 48 h, either alone or in combination with ATRA (1 µmol/L), strongly induced the hyperacetylation of histone H3 and restored RARβ2 expression in HeLa, SiHa, CaSki, and C33A cells. (B) Schematic of the RARβ2-promoter region. The exons of RARβ2 are represented by black boxes. The arrow indicates the transcription-initiation site. The PCR region (−168 to +22) for the ChIP assay included the core region of the RARE, the TATA box, and 22 bp of exon 1. (C) Representative ChIP-PCR data. PCR products were visualized via a 1% agarose gel stained with ethidium bromide. (D) DNA samples from the anti-H3K9ac IP as well as the input material and the mock immunoprecipitation samples were quantified by real-time PCR. Treatment with either 10 µmol/L SAHA or 3 mmol/L VPA led to a significant increase in RARβ2-RARE enrichment. The greatest increase in RARβ2-RARE enrichment was observed with the combination treatment. *P<0.05, **P<0.01.

As shown in Figure 2A, the HDAC inhibitor SAHA (consistent with our previous work on VPA [19]) alone or in combination with ATRA strongly induced hyperacetylation of histone H3 and restored RARβ2 expression in cervical cancer cell lines. To further understand the mechanism for histone acetylation-induced re-expression of RARβ2, we performed a ChIP assay using an anti-H3K9ac antibody to determine the binding of acetylated histone H3 to the RARβ2 promoter region (-168/+22), which includes the core region of the RARE (-55/-35) and the TATA box (Figure 2B). The hyperacetylation of lysine 9 on histone H3 (H3K9ac) is usually associated with actively transcribed genes [23]. Compared to the control group, SiHa cells treated with HDAC inhibitors (10 µmol/L SAHA or 3 mmol/L VPA) alone for 48 h showed a significant increase in the enrichment of RARβ2-RARE based on semi-quantitative (Figure 2C) and real-time PCR (Figure 2D). The effect was the greatest when the cells were treated with both HDAC inhibitors and ATRA (1 µmol/L). This combined treatment was more effective at inducing histone acetylation at the RARβ2-RARE region than any single drug. These results indicate that the RARE in the RARβ2 promoter is functional and that histone acetylation at the RARβ2-RARE region is closely correlated with RARβ2 re-expression in cervical cancer cells.

### Combined treatment of VPA and ATRA inhibits the progression of tumour xenografts derived from human donors

**Figure 3:**
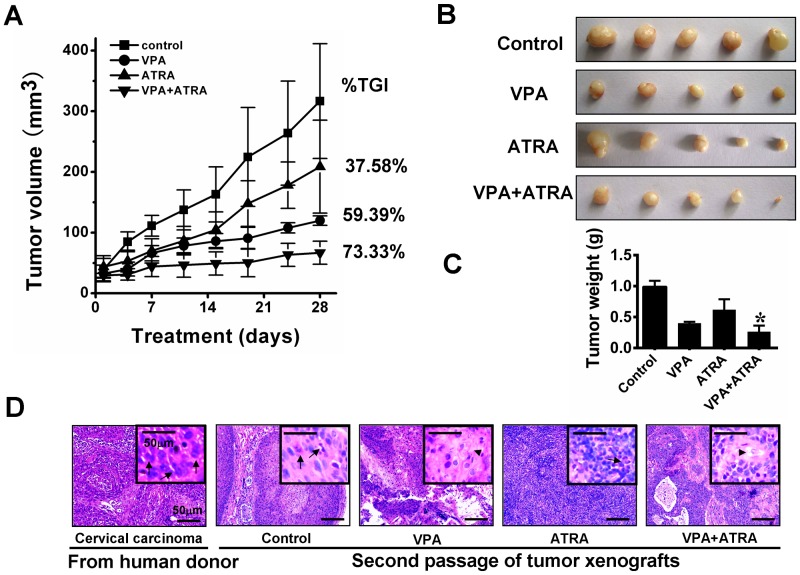
Effect of VPA and ATRA on growth inhibition of tumour xenografts. After the tumors were palpable, the mice were randomly divided into four groups: control (vehicle), VPA (300 mg/kg VPA), ATRA (ATRA 15 mg/kg), and combination. The various treatments were administered daily for 28 days. Tumor volumes and mouse weights were calculated every two days. The combination of VPA and ATRA noticeably inhibited the growth of tumor xenografts compared to either single drug or the vehicle treatment (*P*<0.05) (A). At day 28, the mice were sacrificed, and tumor xenografts were harvested (B). Tumor mass data were consistent with tumor volumes. Greater regression in tumor growth was observed for the combined treatment than for the single-drug administrations (*P*<0.05) (C). VPA and the combination treatment notably improved the degree of histological differentiation. Individual cancer cells had abundant eosinophilic keratinized cytoplasm (arrow heads), and mitotic figures were absent. The tumor xenografts treated with the vehicle or ATRA remained poorly differentiated, similar to the original human tumors. Mitotic figures (arrows) are common in these samples and the cytoplasm is eosinophilic to amphophilic, with minimal or absent keratinization (D). *P<0.05, versus the single-drug treated group.

Four weeks after the initial implantation of the cervical carcinoma derived from a human donor, the tumour became recognisable at the site of transplantation and continued to grow slowly. A rapid growth rate was observed in the second generation; it only took 2 weeks for a noticeable xenograft to form. Histological examination revealed features that were very similar to those of the original patient's cervical carcinoma (Figure 3D).

To determine the effect of VPA and ATRA on tumor xenograft growth, animals received vehicle as a control, VPA (300 mg/kg/d i.p.), ATRA (15 mg/kg/d i.g.), or the combination treatment for 28 days as the tumors were established. The treatment did not have overt toxicity in mice. After 28 days, the mean tumor volume for the combination treatment group was 66.84±19.10 mm^3^, while the tumor volumes for mice treated with vehicle, VPA, and ATRA were 316.65±94.58 mm^3^, 119.83±7.74 mm^3^, and 208.64±76.61 mm^3^, respectively (Figure 3A and B). Consistent with the tumor volume data, the mean tumor weight of the control group was 0.99±0.09 g, while the tumor weights for the VPA, ATRA, and the combination treatment groups were 0.402±0.02 g, 0.618±0.16 g, and 0.264±0.09 g, respectively (Figure 3C). These data indicate that the xenografts were suppressed by 59.39%, 37.58%, and 73.33% (percentage of tumor-growth inhibition, %TGI) for the VPA, ATRA, and combined treatments, respectively (Figure 3A). The data show that the combination treatment resulted in greater growth inhibition of tumor xenografts than either single-drug treatment.

**Figure 4:**
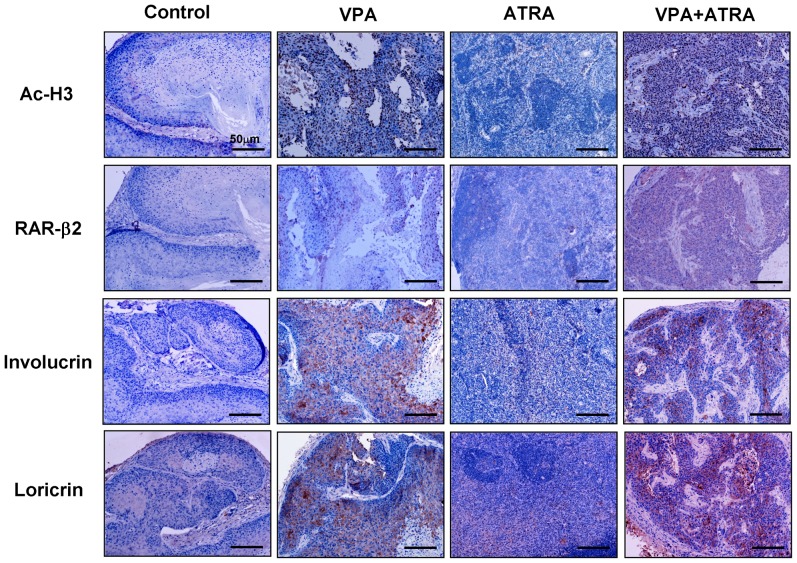
Immunohistochemistry of AcH3, RARβ2, involucrin, and loricrin in tumour xenografts. Treatment with VPA alone significantly increased the level of histone H3 acetylation, restored RARβ2 expression, and induced the expression of the terminal differentiation markers involucrin and loricrin, while treatment with the combination of VPA and ATRA led to greater effect in reactivating these genes expression compared to either single drug treatment. Little positive cells were observed in tissues from the ATRA-treated group.

**Figure 5:**
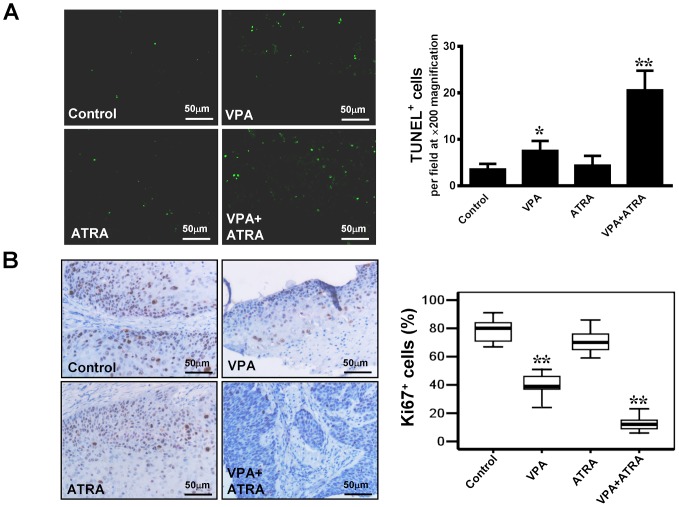
Analysis of cell proliferation and apoptosis in tumor xenografts. (A) Cell apoptosis was evaluated using the TUNEL assay. Apoptotic cells (green) were counted from fluorescence images taken from 10 random fields at 200× magnification. Treatment with the combination of VPA and ATRA significantly increased apoptosis compared to the vehicle or single-drug treatment. (B) Cell proliferation was evaluated using Ki67 staining. The distribution of Ki67-positive cells was scored by two researchers. A significant decrease in the number of Ki67-positive cells was observed in tumors treated with the combination of drugs compared to VPA treatment alone. There was only slightly decrease in the number of Ki67-positive cells in ATRA treated group compared with the control group. *P<0.05, **P<0.01, versus the control group.

Importantly, the histological appearance showed that VPA and the combination treatment improved the extent of histological differentiation in the tumor xenografts. As shown in Figure 3D, cancer cells from xenografts treated with VPA alone or in combination with ATRA have abundant eosinophilic keratinized cytoplasm, while mitotic figures are absent or noted only occasionally. However, cancer cells in the tissues from both the control and ATRA-treated groups were poorly differentiated. Mitotic figures were common (2–4 per high-power field in the control group, but ≤2 per high-power field in ATRA-treated group), and the cytoplasm was eosinophilic to amphophilic, while keratinization was minimal or absent, similar to the original carcinoma. These observations were further verified by immunohistochemical staining for involucrin and loricrin (markers of terminal epithelium differentiation) in tissues from tumor xenografts (Figure 4). The expression of involucrin and loricrin was induced significantly in tumors from mice treated with VPA alone, compared to the tumors from control mice and ATRA-treated mice. The greatest involucrin and loricrin expression levels were found in tumors from the mice treated with the combination of drugs. Cell proliferation was evaluated by Ki67 staining. Treatment with VPA in combination with ATRA led to a significant decrease in Ki67-positive cells compared to VPA, ATRA, or vehicle treatment (13.0% *vs.* 39.4, 71.2, and 78.6, respectively; *P*<0.05) (Figure 5B). In addition to inducing differentiation, VPA also promotes apoptosis [24]. To explore this effect, we performed TUNEL staining to evaluate apoptosis in tumor xenografts (Figure 5A). In tumors from control mice, the number of apoptotic cells was minimal (3.6±1.14). The number of apoptotic cells in tumors from ATRA-treated mice was not significantly different from those in mice treated with VPA alone or the vehicle. There was a significant increase in apoptotic cells (20.60±4.16) in the combination-treated tumors.

### VPA and ATRA restore RARβ2 expression via epigenetic modification and sequentially inhibit tumor growth by reactivating TSGs expression

**Figure 6:**
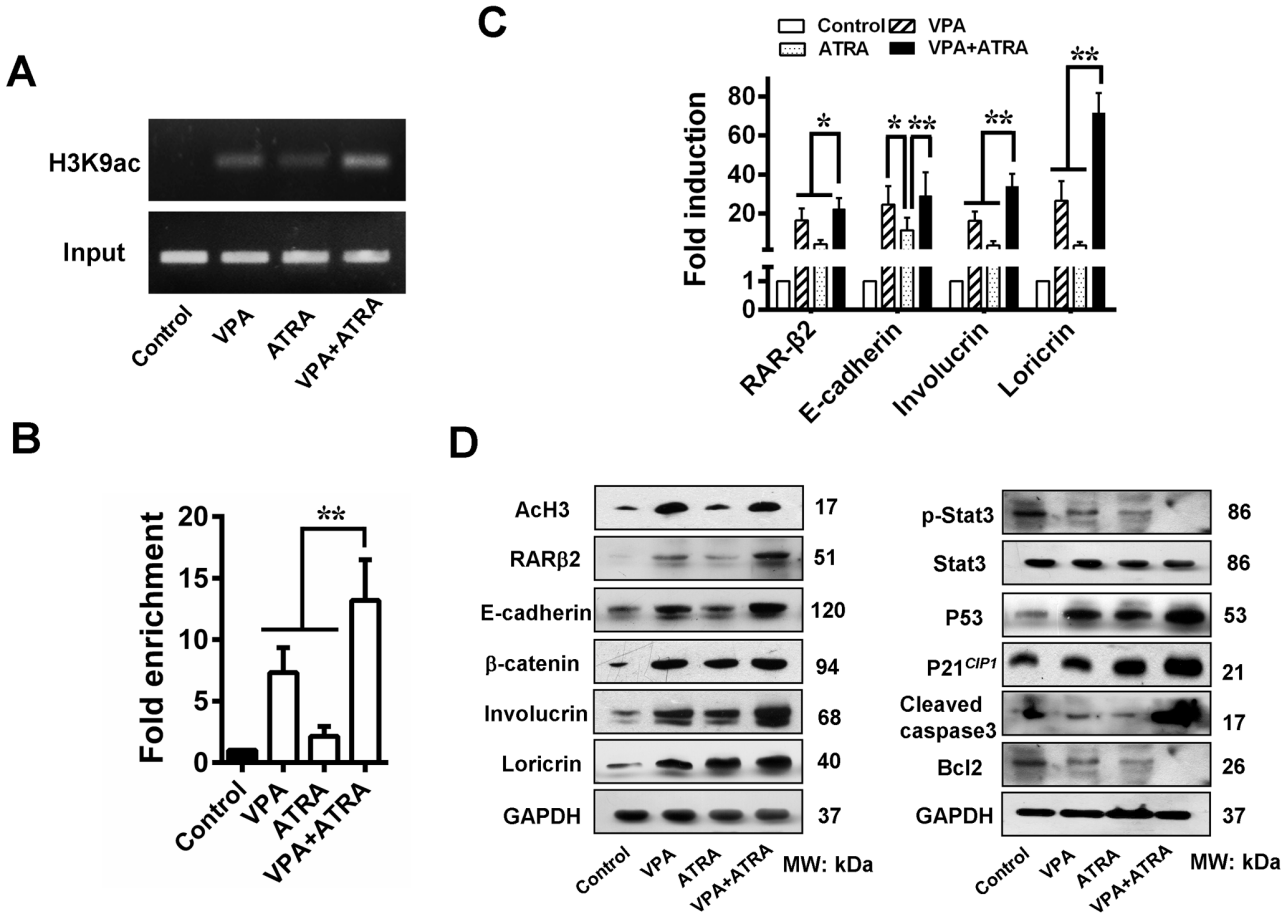
VPA and ATRA promote expression of TSGs. (A) A ChIP assay was used to examine the effect of VPA and ATRA on the level of histone acetylation in the RARβ2-RARE region. (B) A significant increase in RARβ2-RARE enrichment was observed in tumors treated with a combination of VPA and ATRA, indicating that combination treatment restores RARβ2 expression via epigenetic modification. (C) VPA and ATRA restored expression of RARβ2, sequentially enhancing the expression of E-cadherin, involucrin, and loricrin, based on Q-PCR. (D) Immunoblot analysis further showed reactivation of TSGs after treatment with a combination of VPA and ATRA. This treatment resulted in the terminal differentiation and partial apoptosis of the tumour cells in the xenografts. *P<0.05, **P<0.01.

The histone H3 acetylation status of the tumour xenografts was determined using immunohistochemistry and Western blot analysis. As shown in Figures 4 and 6, VPA treatment significantly increased the level of acetylated H3 compared to the control and the ATRA group. When combined with ATRA, VPA exhibited an evident additive effect in histone epigenetic modification. To investigate the effect of these reagents on the level of histone acetylation at the RARβ2-promoter region, a ChIP assay was performed. We observed ∼7-fold and ∼2-fold increases in RARβ2-RARE enrichment for the VPA- and ATRA-treated tumors, respectively, whereas the greatest effect was observed in the combination-treated tumors (∼13-fold) (Figure 6A and B). Therefore, the combination treatment reactivated RARβ2 expression via epigenetic modification, sequentially enhancing the expression of target genes, including E-cadherin, involucrin, and loricrin (Figures 4 and 6). The transcript levels for the functional retinoic pathway further verified the existence of an additive effect (Figure 6C). Furthermore, a concomitant increase in the levels of P21*^CIP1^*, P53, and activated caspase 3 as well as a decrease in the levels of p-Stat3 and Bcl2 were noted (Figure 6D). These results indicate that HDAC inhibitors and ATRA reactivate RARβ2 via histone modification and sequentially upregulate other TSGs, thereby restoring the function of key regulatory pathways for cell differentiation and apoptosis, which contribute to the inhibition of tumor xenografts.

## Discussion

Epigenetic silencing of TSGs is a salient feature of tumour cells. Recent studies have indicated that aberrant HDAC activity disturbs normal epigenetic processes through inappropriate deacetylation, thereby leading to euchromatin gene silencing [1]–[3], [7], [25]. However, the association between histone acetylation, TSG expression, and clinicopathological parameters in human cervical cancer is poorly understood. Therefore, we designed this study to quantify the level of acetylated histone H3 and the aberrant expression of RARβ2 and the E-cadherin/β-catenin complex in biopsies of cervical squamous cell carcinoma patients using immunohistochemistry, to determine the relationship between the expression levels of these markers and the clinicopathological features of the patients, and to evaluate the therapeutic efficacy of the HDAC inhibitor VPA combined with ATRA by targeting chromatin remodelling and RARβ expression.

The results of this study show a positive correlation between histone H3 acetylation and the TSG expression of RARβ2 and E-cadherin in cervical squamous cell carcinoma specimens. Recent studies have verified that the combination of local histone deacetylation and CpG island methylation results in the strong epigenetic repression of RARβ2 and E-cadherin and, therefore, resistance to the growth inhibitory effects of retinoic acid [4], [26], [27]. Additionally, as it is downstream of retinoid signalling, the reduced expression of RARβ2 also results in the loss of E-cadherin in some cancer types [19], [28], [29]. Consistent with this observation, we also showed a direct correlation between RARβ2 and E-cadherin expression in cervical tumours.

Moreover, in this study, the degree of AcH3 expression displayed a significant association with histological differentiation and nodal metastasis, but there was no difference with respect to age or clinical stage. Accumulating evidence has shown that RARβ2 is the principal mediator of retinoid differentiation and antiproliferative effects in epithelial tumour cells [12], [19], [30]. E-cadherin-mediated adhesion plays an essential role in epidermal cell differentiation via the PI3K/Akt pathway by binding to β-catenin [19], [31], whereas the ablation of E-cadherin impairs both the survival and the differentiation of epidermal keratinocytes [32], [33]. Taken together, these results suggest that histone acetylation is required for the transcription of RARβ2 and E-cadherin and thus regulates histological differentiation in cervical tumours. However, as previous studies indicate, the aberrant acetylation of histones on the RARβ2 promoter, especially at the core region of the RARE, results in decreased or silenced RARβ2 expression in many cancer types, including cervical cancer [9], [18], [19]. The histone deacetylase complex strictly binds to RARs to inhibit transcription of RA-responsive genes under steady-state conditions [34]. Thus, it is essential for RARβ2 transactivation to increase the acetylation of histones on RARE through HDAC inhibitors. Our ChIP assay showed that HDAC inhibitors (SAHA and VPA) alone or in combination with ATRA markedly increased the enrichment of acetylated histones (H3K9ac) on the RARβ2-RARE region both *in vitro* and *in vivo*, leading to a restoration of RARβ2 and the upregulation of its downstream growth inhibitory pathways in cervical cancer.

Indeed, a number of HDAC inhibitors are therapeutically effective on a variety of hematological and solid tumors both *in vitro* and *in vivo*, including leukemia, lymphoma, lung cancer, breast cancer, melanoma, glioblastoma, uterine cervix cancer, among others [7], [10], [18], [19], [35], [36]. Moreover, a number of inhibitors are undergoing clinical trials [37]–[41]. However, as single agents, these molecules have limited activity, with the exception of the treatment of cutaneous T-cell lymphoma (CTCL). In cervical cancer, some HDAC inhibitors, such as VPA, SAHA, BML-210, and trichostatin A (TSA), significantly inhibit tumor growth when combined with DNA-demethylating agents, proteosome inhibitors, or cisplatin. The combined treatments are superior to HDAC-inhibitor treatment alone [19], [38], [42], [43]. It is likely that broad efficacy of HDAC inhibitors will only be realized in combination with other drugs. ATRA is a potential regulator of normal cell development, growth, and differentiation [12]. Sequential treatment with HDAC inhibitor and ATRA led to reprogrammed differentiation for acute myeloid leukemia and neuroblastoma [44], [45]. A recent phase I clinical trial of the HDAC inhibitor entinostat in combination with 13-cis retinoic acid in 19 patients with solid tumors revealed that the combination treatment was effective at controlling disease progression, although no tumor responses were observed [46]. Our latest report also indicates that VPA restores RARβ2 expression and has a significant synergistic effect on growth inhibition when combined with ATRA in cervical cancer cell lines [19]. Furthermore, in the present study, we investigated the anti-cancer effects of VPA in combination with ATRA in a xenograft model in which nude mice were transplanted with poorly differentiated human squamous cell carcinoma. It is believed that these transplanted human tumors represent good models for experimental oncostatic therapeutic research [47], [48]. Our results show that tumors transplanted in the nude mice maintain their histological resemblance to the original primary tumor after the second generation. Consistent with previous findings [18], [19], [35], VPA and ATRA administered at their therapeutic concentrations exert an evident additive antitumor effect (TGI 73.33%) on xenografts that is superior to the effect of either drug alone. VPA alone also exhibited significant tumor repression (TGI 59.39%). However, it is worthwhile to note that the tumor xenografts were suppressed by 37.58% with ATRA alone (15 mg/kg/day), although the tumor lacked RARβ2 expression. Various mechanisms are most likely involved in the tumor-repression process. Previous studies have shown that ATRA upregulates RARβ expression [49] and interacts with other transcription factors in addition to RAR/RXR heterodimers to mediate gene expression [50]. In the present study and in our previous work [19], RARβ2 was restored both *in vitro* and *in vivo* compared with the control group. There is an interesting report that P21*^CIP1^*, which also contains the RARE in the promoter region, can transcriptionally activate the upstream promoter region of RARβ [51]. In addition, the physiological ligand ATRA can elicit transcriptional effects independent of binding to RARs. For example, this process can occur through the peroxisome proliferator-activated receptor (PPAR) δ [52], [53].

Mechanistically, treatment with VPA and ATRA significantly increased the level of histone H3 acetylation, reactivated RARβ2 and E-cadherin expression, and upregulated P21*^CIP1^* and P53, whereas this treatment downregulated phosphorylated Stat3. More importantly, the combination treatment notably increased the expression of involucrin and loricrin, two late differentiation markers of keratinocytes. Therefore, we suggest that the regression of the xenografts treated with VPA and ATRA results primarily from differentiation and partially from apoptosis.

In conclusion, the decrease in the level of histone acetylation was closely associated with the progression of cervical cancer through the silencing of TSGs. Targeted therapy with HDAC inhibitors and RARβ2 agonists can restore pivotal TSG expression and produce significant antitumour effects by promoting differentiation in cervical squamous cell carcinoma. These results pave the way for the clinical testing of differentiation therapy in cervical cancer.
